# Gold Nanostars-AIE Theranostic Nanodots with Enhanced Fluorescence and Photosensitization Towards Effective Image-Guided Photodynamic Therapy

**DOI:** 10.1007/s40820-020-00583-2

**Published:** 2021-01-16

**Authors:** Mohammad Tavakkoli Yaraki, Min Wu, Eshu Middha, Wenbo Wu, Soroosh Daqiqeh Rezaei, Bin Liu, Yen Nee Tan

**Affiliations:** 1 Institute of Materials Research and Engineering, Agency for Science, Technology and Research (A*STAR), Singapore, 138634 Singapore; 2 Department of Chemical and Biomolecular Engineering, National University of Singapore, 4 Engineering Drive 4, Singapore, 117585 Singapore; 3 Department of Mechanical Engineering, National University of Singapore, 9 Engineering Drive 1, Singapore, 117575 Singapore; 4 Faculty of Science, Agriculture and Engineering, Newcastle University, Newcastle Upon Tyne, NE1 7RU UK; 5 Newcastle Research and Innovation Institute (NewRIIS), 80 Jurong East Street 21, #05-04, Singapore, 609607 Singapore

**Keywords:** Plasmon enhancement, Photosensitizer, Aggregation-induced emission, Fluorescence imaging, Theranostics

## Abstract

Dual-functional aggregation-induced photosensitizers (AIE-PSs) with singlet oxygen generation (SOG) ability and bright fluorescence in aggregated state have received much attention in image-guided photodynamic therapy (PDT). However, designing an AIE-PS with both high SOG and intense fluorescence via molecular design is still challenging. In this work, we report a new nanohybrid consisting of gold nanostar (AuNS) and AIE-PS dots with enhanced fluorescence and photosensitization for theranostic applications. The spectral overlap between the extinction of AuNS and fluorescence emission of AIE-PS dots (665 nm) is carefully selected using five different AuNSs with distinct localized surface plasmon (LSPR) peaks. Results show that all the AuNSs can enhance the ^1^O_2_ production of AIE-PS dots, among which the AuNS with LSPR peak at 585 nm exhibited the highest ^1^O_2_ enhancement factor of 15-fold with increased fluorescence brightness. To the best of our knowledge, this is the highest enhancement factor reported for the metal-enhanced singlet oxygen generation systems. The Au585@AIE-PS nanodots were applied for simultaneous fluorescence imaging and photodynamic ablation of HeLa cancer cells with strongly enhanced PDT efficiency in vitro. This study provides a better understanding of the metal-enhanced AIE-PS nanohybrid systems, opening up new avenue towards advanced image-guided PDT with greatly improved efficacy.

**Supplementary Information:**

The online version of this article (10.1007/s40820-020-00583-2) contains supplementary material, which is available to authorized users.

## Highlights

- Development of Au nanostar@AIE nanodots photosensitizers for theranostic applications.
- Achieving excellent metal-enhanced fluorescence and singlet oxygen generation efficiency simultaneously.
- Mechanism study of the role of FRET on metal-enhanced singlet oxygen generation.

**Supplementary Information:**

The online version of this article (10.1007/s40820-020-00583-2) contains supplementary material, which is available to authorized users.

## Graphical abstract

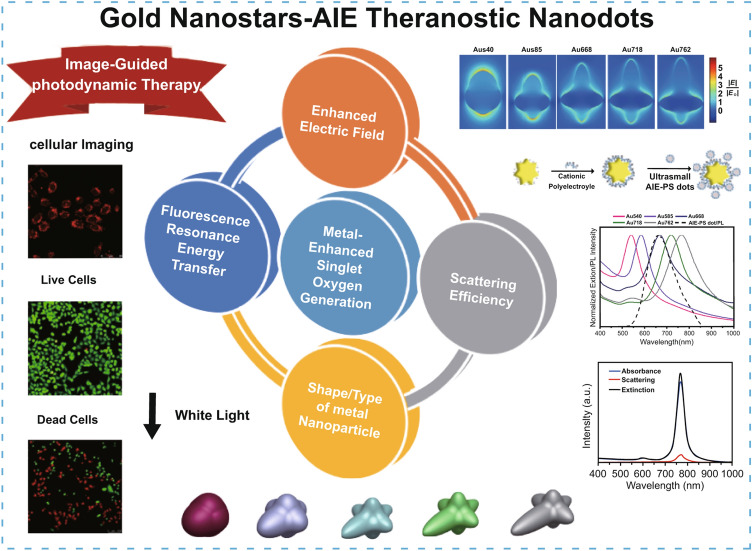

## Introduction

Photodynamic therapy (PDT) has emerged as a non-invasive method for cancer treatment in recent years. PDT relies on the absorption of light by the photosensitizer molecules and energy transfer from the excited photosensitizer molecule to nearby oxygen and water molecules to produce reactive oxygen species (ROS) such as peroxides, superoxide, hydroxyl radical, singlet oxygen [1, 2]. In particular, the electrons in the triplet state of most of photosensitizers can be transferred to the surrounding oxygen molecules to produce singlet oxygen (^1^O_2_) in killing cancer cells [3]. Singlet oxygen, which is the lowest excited state of molecular oxygen, is the most stable and well-known cytotoxic agent owing to its high reactivity [4, 5]. The efficiency of PDT is determined by the production of sufficient ^1^O_2_ molecules that could be obtained by delivering a high dosage of the photosensitizers to the targeted tissue/cells [6]. Recently, the progress in PDT has witnessed the development in image-guided PDT, where an efficient therapy with bright fluorescence ability could be used for tracking and visualization of photosensitizers inside the tumour, allowing real-time monitoring of the therapeutic outcome [7–11]. However, classical organic photosensitizers, e.g. methylene blue, suffer from self-degradation under light irradiation and aggregation at high concentrations due to π–π stacking, resulting in a reduction in their fluorescence and singlet oxygen generation (SOG) ability [12]. To address this drawback, a new generation of photosensitizer, termed as aggregation-induced emission photosensitizer (AIE-PS), has been developed [13–16]. These AIE photosensitizers are not emissive in the molecular state, but become fluorescent in the aggregate state as well as possessing intrinsic ability of SOG with high photostability under light illumination [17–20].

For image-guided therapy, it is desirable to use the fluorescent PSs with sufficient brightness for imaging, at the same time generating ROS with high efficiency for better PDT performance. It would also allow the use of a lesser amount of photosensitizer so that biosafety can be further improved [21–23]. However, it is challenging to improve the brightness of fluorescence and efficiency of SOG concurrently via molecular design as both functions are competing for the excited energy. The plasmonic properties of metal nanoparticles (NPs) not only can be used to enhance the signal of fluorophores or sensing molecules [24–27], but also the intrinsic SOG ability of photosensitizers. It was reported that by placing the AIE-PSs in vicinity of the metal NPs such as gold (Au) and silver (Ag), the SOG efficiency of AIE-PS can be enhanced by several folds [15, 16]. This metal-enhanced SOG approach (ME-SOG) has been successfully developed and employed for antimicrobial photodynamic inactivation of micro-organisms (PDI) [5] and photodynamic cancer therapy [28]. Among the developed ME-SOG systems, the highest SOG enhancement factors have been reported for the silver nanospheres (i.e. tenfold using 85-nm AgNPs-enhanced AIE photosensitizer with a final SOG efficiency of 2.35) [16] and silver nanocubes (i.e. fourfold using 80-nm Ag@Silica nanocube-enhanced Rose Bengal with a final SOG efficiency of 3) [29] owing to the substantial enhanced electric field at the surface of the nanosilver. However, these Ag nanoparticles have shown considerable dark cytotoxicity against normal cells [30, 31]. Since the PDT efficiency is directly proportional to the concentration of metal@photosensitizer nanohybrids, the cytotoxicity of Ag nanoparticles at high concentrations restricts their in vivo PDT applications. Hence, developing ME-SOG systems based on less toxic materials such as gold is of interest for therapeutic applications. In addition, fundamental understanding of the design parameters such as composition [32], size [33], and shape [29] of metal nanoparticles, intrinsic singlet oxygen quantum yield of photosensitizers [34], as well as the degree of spectral overlap between the extinction of metal nanoparticles and absorbance of photosensitizers play important roles on the performance of colloidal ME-SOG system. However, most of the previously reported colloidal ME-SOG systems either have not studied the fluorescence phenomenon [32, 35–38] or have observed fluorescence quenching for the ME-SOG samples. [15, 16] Indeed, simultaneous enhancement in both SOG and brightness of fluorescence photosensitizer, which is important for image-guided therapy, has rarely been reported [39].

In this study, we reported new gold nanostars-enhanced AIE theranostic nanodots with greatly improved singlet oxygen generation and fluorescence brightness towards effective image-guided photodynamic therapy. Specifically, the effect of spectral overlap between the absorbance of metal NP and fluorescence of red-emissive AIE-PS has been investigated experimentally and theoretically on metal-enhanced singlet oxygen generation. To study this effect, five different sizes of positively charged gold nanostars (AuNS) with distinct localized surface plasmon resonance (LSPR) peaks ranging from 540 to 762 nm have been successfully synthesized. The red-emissive AIE-PS was formulated into negatively charged nanodots (5 nm in diameter) using microfluidics. Subsequently, the two oppositely charged AuNS and AIE-PS nanodots were mixed in an appropriate ratio and concentrations to form the metal-enhanced SOG nanohybrid system. A hitherto unreported SOG enhancement factor of 15-fold was obtained by using the AuNS with LSPR peak at 585 nm (i.e. Au585) and AIE-PS nanodots with emission wavelength at 665 nm. Simulation study was also conducted to confirm that the enhanced electric field for Au585 is higher than the other AuNSs studied herein. Moreover, the fluorescence intensity of all nanohybrid samples showed enhancement instead of quenching as compared with the control sample (i.e. AIE-PS dot). Cytotoxicity test showed that these AuNS@AIE-PS nanodots possess considerable biocompatibility with both normal and cancerous cells under dark condition. Finally, the Au585@AIE-PS nanodots were applied for simultaneous fluorescence imaging and photodynamic ablation of HeLa cancer cells, achieving a stronger PDT efficiency as compared to the control (i.e. AIE-PS dots of similar concentration) as well as showing bright red fluorescence in cellular imaging. These results demonstrated the promising potential of the metal-enhanced AIE photosensitizer nanodots towards advanced image-guided photodynamic therapy with greatly improved efficiency and sensitivity for theranostic treatment.

## Experimental Section

### Chemicals

Gold(III) chloride trihydrate (HAuCl_4_.3H_2_O), (4-(2-hydroxyethyl)-1-piperazineethanesulfonic acid) (HEPES buffer), polyethyleneimine (PEI, 50% in water, Mw = 2000 Da), 1,2-distearoyl-sn-glycero-3-phosphoethanolamine-N-(methoxy(polyethylene glycol)-2000) (DSPE-mPEG2000), 9,10-anthracenediyl-bis(methylene)dimalonic acid (ABDA), Rose Bengal, Methylene Blue, Verteporfin, sodium hydroxide, hydrochloric acid (37%), and tetrahydrofuran (THF) were purchased from Sigma-Aldrich and used as received. Ultrapure water (18.2 MΩ·cm) was used to prepare all aqueous solutions.

### Preparation of Ultrasmall AIE-PS Nanodots Using Microfluidic Nanoprecipitation Method

The AIE-PS of TPATCN was synthesized according to our previous work [40]. The ultrasmall AIE-PS dots (about 5 nm) of TPATCN were prepared using the microfluidics nanoprecipitation method at a high Reynolds number (Re = 350). DSPE-mPEG2k was used as a polymer to encapsulate the organic AIE-PS in this study. Details of the synthesis procedure can be referred to Scheme S1 in Supporting Information (SI-1, page S1).

### Synthesis of PEI-Capped Au Nanostars (AuNS)

Five different Au nanostars with localized surface plasmon peak between 590 and 762 nm were synthesized according to the literature [41]. Briefly, 30 mL aqueous solution of HEPES buffer solution was prepared, and its pH was adjusted at 7.2. Then, the HEPES solution was stirred for 1 min (400 rpm) followed by addition of 600 µL of HAuCl_4_ solution (20 mM). The reaction vessel was stirred for 30 min following overnight keeping in dark condition. To synthesize Au nanostars (AuNSs) with different LSPR peak, the HEPES/Au ratio (R) was adjusted accordingly, i.e. R = 200 (for Au540), 350 (for Au585), 600 (for Au668), 750 (for Au718), and 900 (for Au762), respectively. The as-synthesized AuNSs were washed by centrifugation at 10,000 rpm (three times) to remove the excess HEPES molecules and dispersed in 10 mL of DI-water (pH 6) prior to surface modification with PEI. To make PEI-capped AuNSs, 10 mL of as-synthesized AuNSs was transferred to a round bottle flask, followed by addition of 5 mL of PEI solution (1 mg mL^−1^, pH 6). The mixture solution was stirred vigorously for 3 h to complete the adsorption process. Finally, the PEI-capped AuNSs were washed by DI-water and re-dispersed in 10 µL DI-water. The concentration of each PEI-capped AuNSs was estimated according to the extinction coefficient reported in the literature [42]. For the convenience of discussion, the five PEI-capped AuNS samples are referred to as Au540, Au585, Au668, Au718, and Au762 hereafter for Au nanostars with surface plasmon band at 540, 585, 668, 718, and 762 nm, respectively.

### Detection of Singlet Oxygen (^1^O_2_) Generation

The singlet oxygen generation rate was measured by a chemical trapping method using ABDA as a singlet oxygen selective indicator. 1 mL solution of AuNS@AIE-PS with a final AIE-PS concentration of 2 µg mL^−1^ was transferred to a 1-mL disposable cuvette. The sample was irradiated by 30 mW cm^−2^ white light in the presence of ABDA (final concentration of 50 µM). The absorbance of the sample was measured every 5 min, and the rate of degradation of ABDA was calculated using the following first-order kinetic model [43]:

1 $$\ln \left( {\frac{A\left( t \right)}{{A\left( 0 \right)}}} \right) = - k.t$$

where *A(t)* and *A(0)* are absorbance of ABDA at 379 nm at time t and 0, respectively. *k* is the first-order kinetic constant for degradation of ABDA and *t* is time. The absorbance of AIE-PS dots (at 379 nm) in each sample was subtracted from the observed absorbance of the ABDA before calculation of the degradation rate.

### ^1^O_2_ Quantum Yield Measurements

^1^O_2_ quantum yield was measured using ABDA as an indicator for singlet oxygen generation. Water-soluble Rose Bengal was selected as a reference photosensitizer. Briefly, 1 mL of aqueous solution containing AIE-PS dot ( 10 µg mL^−1^) or 10-µM Rose Bengal, and 50-µM ABDA was prepared and subjected to white light (55 mW cm^−2^) irradiation at the same conditions as aforementioned. The degradation rate of ABDA was monitored by measuring its absorbance intensity at 379 nm under irradiation over time. The ^*1*^*O*_*2*_ quantum yield was calculated using equation [9]:

2 $$\varphi = \varphi_{{{\text{RB}}}} \times \frac{{k_{{\text{AIE - PS dot}}} }}{{k_{{{\text{RB}}}} }} \times \frac{{A_{{{\text{RB}}}} }}{{A_{{\text{AIE - PS dot}}} }}$$

where $k_{{\text{AIE - PS dot}}}$ and $k_{{{\text{RB}}}}$ are degradation rate of the ABDA by the AIE-PS dot sample and Rose Bengal, respectively. $A_{{\text{AIE - PS dot}}}$ and $A_{{{\text{RB}}}}$ are integral of absorbance spectra data for the AIE-PS dot sample and Rose Bengal in the wavelength range of 400–800 nm. $\varphi_{{{\text{RB}}}}$ is ^*1*^*O*_*2*_ quantum yield of Rose Bengal in water, which is 75% [44].

### Time-Resolved Fluorescence Spectroscopy

The as-prepared AuNS@AIE-PS samples were excited at 488 nm, and their fluorescence decay profiles were collected at 665 nm using a FluoTime 200 TCSPC machine (Picoquant BmbH, Germany) for fluorescence lifetime measurement. The below two-exponential model was used to model the fluorescence decay profiles [45].

3 $$I\left( t \right) = \sum \alpha_{i} \exp ( - t/\tau_{i} )$$

where $I\left( t \right)$ is fluorescence intensity at time t, $\tau_{i}$ is decay time, $\alpha_{i}$ is amplitude, and $\sum \alpha_{i} = 1$.

The average fluorescence lifetime can be calculated using the below equation:

4 $$\overline{\tau } = \sum f_{i} \tau_{i}$$

where $f_{i}$ is the contribution fraction of each component and is defined as the following equation:

5 $$f_{i} = \frac{{\alpha_{i} \tau_{i} }}{{\sum \alpha_{i} \tau_{i} }}$$

The curve fitting was performed using the curve-fitting toolbox in MATLAB2014b software.

### Characterizations

UV–visible absorption and fluorescence spectra were recorded using a Shimadzu UV-2450 spectrophotometer and an Infinite M-200 microplate reader, respectively. TEM images were obtained on a JEOL JEM-2010F transmission electron microscopy operating at 200 kV. The zeta potential and hydrodynamic size of the gold nanostars were acquired on Malvern Zetasizer at room temperature. The Fourier-transform infrared (FTIR) spectroscopy was carried out using a VERTEX 70/70 v FTIR spectrophotometer with an attenuated total reflectance (ATR) mode in the range of 500–4000 cm^−1^.

### Confocal Imaging

HeLa cells were cultured in an eight-well chamber (LAB-TEK, Chambered Coverglass System) at 37 °C in a humidified environment containing 5% CO_2_. After reaching 80% confluence, the cell culture medium was replaced with fresh culture medium containing AIE dot or Au585@AIE-PS at AIE photosensitizer concentration of 10 μg mL^−1^. After 12 h of incubation, the cells were washed three times with PBS (1X), and their nuclei were stained by Hoechst for 5 min. After washing the cells, they were immediately imaged by a confocal laser scanning microscope (CLSM). The red emission from AIE dots (600–680 nm) was collected by excitation at 488 nm. The blue emission from Hoechst (430–470 nm) was acquired between upon excitation at 405 nm. The obtained images were analysed by ImageJ software.

### Cell Viability and Cell Ablation Study

The viabilities of normal mammalian cells (NIH-3T3) and cancer cells (HeLa) were evaluated using methylthiazolyldiphenyltetrazolium bromide (MTT) assays for the cells incubated with the samples with different concentrations of samples before and after photodynamic therapy. First, NIH-3T3 and HeLa cells were seeded in 96-well plates (Costar, IL, U.S.A.) at an intensity of 4 × 10^4^ cells mL^−1^. After 24-h culturing, the cells were incubated with AIE-dots, Au585@AIE-PS, Au585, and Verteporfin in DMEM suspension at various concentrations for 12 h. Then, the suspension was replaced by the fresh cell culture medium. The selected samples were treated by white light irradiation (100 mW cm^−2^ and 10 min ~ 60 J cm^−2^) and further cultured for 24 h. The control samples were considered the same samples without light treatment and incubation in the dark condition. Next, all cells incubated with freshly prepared MTT solution (0.5 mg mL^−1^, 100 μL well^−1^) for 3 h. After removing the MTT solution, 100 μL of filtered DMSO was added into each well to dissolve all the crystals formed. The cell viability was accessed using MTT absorbance at 570 nm recorded using a Microplate reader (Genios Tecan). The cells incubated with culture medium only (without the addition of any AIE-dots, Au585@AIE-PS, Au585, or Verteporfin samples) were designated as cells with 100% cell viability.

### Live/Dead Cell Staining

The HeLa cells were seeded in an eight-well chamber. After reaching 80% confluence, the cell culture medium was replaced by fresh culture medium-containing AIE dot or Au585@AIE-PS (10 μg mL^−1^ based on AIE photosensitizer). After 12 h of incubation, the cells were washed and replaced with fresh medium. Selected cells were treated with light irradiation for a designated time (0, 5, and 10 min) and light power (100 mW cm^−2^). After light treatment, the cells were then incubated with fluorescein diacetate (10 μg mL^−1^) and PI (10 μg mL^−1^) for 20 min each in sequence. After washing, the cells were imaged by CLSM. The excitation wavelength was set at 488 and 543 nm to collect the green fluorescence from fluorescein diacetate (510–520 nm) and the red fluorescence from PI (600–680 nm), respectively.

### Finite Element Method Simulation

In order to calculate the electric field, and extinction efficiency of AuNSs, finite element method (FEM) simulations were carried out in COMSOL Multiphysics software package (see www.comsol.com). The complex refractive index of Au was taken, Johnson and Christy data [46]. Water was used as the surrounding environment (*n* = 1.33). A plane wave propagating in the *x*-direction and linearly polarized along the *z*-axis spanning the wavelength range of 400–1000 nm was set as the source. The dimensions of each gold nanostar were extracted from TEM images, and then, the extinction spectra for each gold nanostar were modelled according to the literature [42]. The dimensions used to model each nanostar and the predicted LSPR peaks for different nanostars are shown in Fig. S5 and Table S1. The simulated spectra for each nanostar are shown in Fig. S6. A fillet with 5 nm radius was used to round the sharp junctions and corners in all samples.

## Results and Discussion

### Synthesis and Characterizations of AIE Photosensitizer Nanodots

**Fig. 1:**
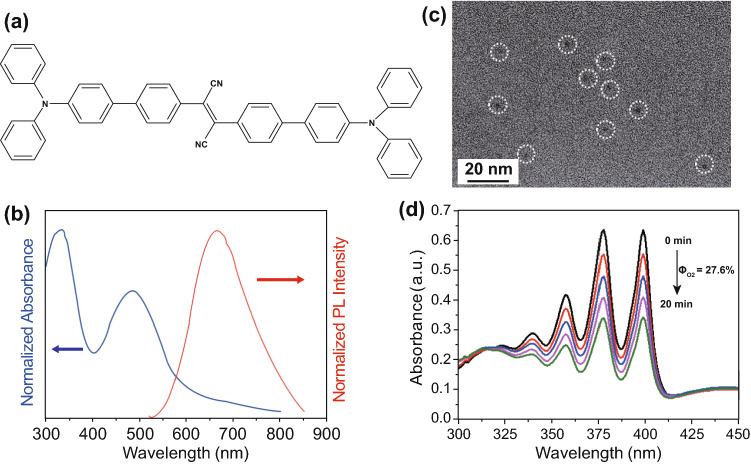
**a** Chemical structure of TPATCN as AIE-PS. **b** Normalized UV–Visible absorption and fluorescence spectra (*λ*_ex_ = 490 nm) of AIE-PS nanodots. **c** TEM images of the AIE-PS nanodots encapsulated by DSPE-PEG-2000. **d** Degradation of 50 μM ABDA in the presence of 10 μg mL^−1^ AIE-PS dots under white light irradiation

In this study, 2,3-bis(4′-(diphenylamino)-[1,1′-biphenyl]-4-yl)fumaronitrile (TPATCN) was used as a model aggregation-induced emission photosensitizer (AIE-PS) (Fig. 1a). This molecule shows broad absorption spectra with two main peaks at 333 and 490 nm, as well as near-infrared fluorescence emission in the aggregated state, cantered at 665 nm (Fig. 1b) with high photostability [47, 48]. In the molecular design, triphenylamine (TPA) is used as the donor, while fumaronitrile (FN) is used as the acceptor. The phenyl rings between the donor and acceptor can separate the HOMO–LUMO distribution. The HOMO–LUMO separation promotes the intersystem crossing rate, resulting in favourable singlet oxygen generation [14, 40]. In this study, ultra-small AIE-PS nanodots with good uniformity are formed by encapsulating the TPATCN molecules into the DSPE-mPEG polymer via the microfluidic nanoprecipitation. This method could produce nanodots with high reproducibility and monodispersity. (See details of the microfluidic approach in Scheme S1, Supporting information monodispersity [49–52].) As rapid mixing of solvent with anti-solvent (water) results in the instantaneous formation of nanodots due to the high level of supersaturation of the precursors [53, 54], we performed the synthesis at a high flow rate of 16.5 mL min^−1^ for AIE-PS/polymer mixture solution with a mass ratio of 2 and Reynolds number of 350 (Fig. S1). This rapid mixing of organic solvent into the anti-solvent (aqueous phase) causes the spontaneous formation of the ultra-small AIE-PS nanodots by taking advantage of the highest Reynolds (Re). Figure 1c shows the transmission electron microscopy (TEM) images of AIE-PS nanodots encapsulated in the DSPE-mPEG2000. Under the fabrication conditions, the polymers exhibited negative charges leading to the formation of AIE-PS nanodots with an average size of 5.0 ± 1.0 nm and zeta potential of -38.0 ± 2.3 mV. This is due to the exposure of hydroxyl groups of the PEG2000 on the surface of the PEI-modified AIE-PS dots. While maintaining its fluorescence properties, the as-obtained AIE-PS nanodots also produce singlet oxygen under white light with a SOG quantum efficiency of 27% (using Rose Bengal as reference), monitored by 9,10-anthracenediyl-bis(methylene)dimalonic acid (ABDA) as a specific ^1^O_2_ indicator (Fig. 1d and Scheme S2).

### Formation of Au Nanostars@AIE-PS Nanodots and Their Singlet Oxygen Generation Properties

**Fig. 2:**
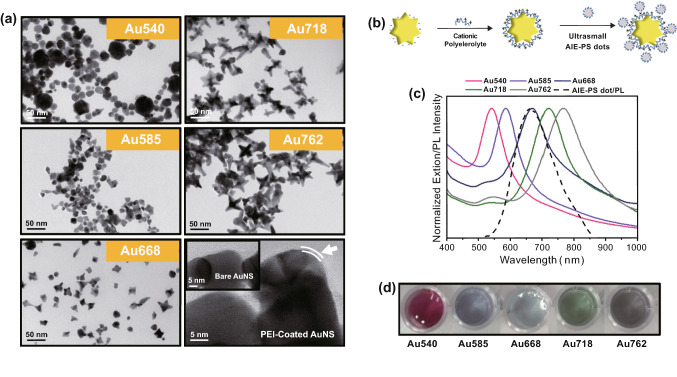
**a** TEM images of the as-synthesized AuNSs with different LSPR peaks as labelled in each image. The last image indicates the existence of a very thin layer of PEI on the surface of AuNSs after surface modification with (indicated by two parallel lines), while the inset shows the TEM image of bare AuNS. **b** Schematic illustration of the AuNS@AIE-PS formation via electrostatic interaction. **c** Spectral overlap between the extinction spectra of different AuNSs (solid lines) and fluorescence spectra of AIE-PS dot (dashed line). **d** Solution colours of the as-synthesized AuNSs with LSPR peak at 540, 585, 668, 718, and 762 nm (left to right), respectively, under room light

For the investigation of metal-enhanced singlet oxygen generation (ME-SOG), five sets of gold nanostars (AuNSs) with distinct LSPR peak in the range of 540–762 nm were synthesized. The morphology and surface charge of the as-synthesized AuNSs were measured by TEM and Zetasizer, respectively. As shown in Fig. 2, all the AuNSs showed uniform size distribution where their LSPR peak wavelength experiences red-shift with increasing length of the AuNS branches. These negatively charged AuNSs were then functionalized with polyethyleneimine (PEI) molecules, resulting in the formation of positively charged PEI-capped AuNSs (see their hydrodynamic diameter, zeta potential, and FTIR spectra in Fig. S2a-c). These positively charged AuNSs were then mixed with the negatively charged AIE-PS nanodots in aqueous solution of pH 4 to form the AuNS@AIE-PS nanohybrids (Figs. 2b and S2d). Both the as-synthesized AIE-PS dots and AuNS@AIE-PS nanohybrids showed good colloidal stability under storage conditions of 4 °C for at least 3 weeks (Fig. S2e).

**Fig. 3:**
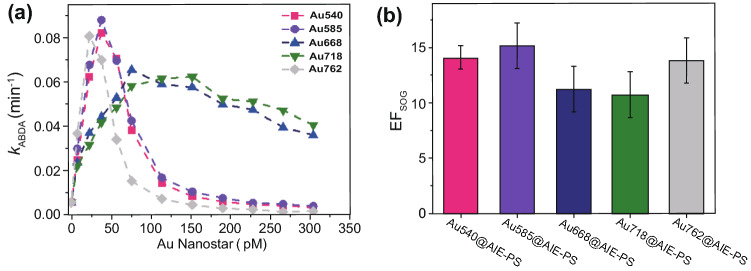
**a** Plot of kinetic constant degradation of ABDA (*k*_ABDA_) versus different concentration of AuNSs showing the plasmonic enhancement effect of Au540 (pink), Au585 (violet), Au668 (blue), Au718 (green), and Au762 (grey), respectively, on the singlet oxygen generation (SOG) efficiency of AIE-PS nanodots (2 μg mL^−1^). **b** Calculated SOG enhancement factor (*EF*_SOG_) for the AuNS@AIE-PS nanohybrids at the respective critical concentration of AuNS at maximum SOG. 30 mW cm^−2^ white light was used for all the SOG experiments

To monitor the rate of singlet oxygen generation (SOG) in the AuNS@AIE-PS samples, ABDA was used as the selective ^*1*^*O*_*2*_ trapping probe. The degradation rate of ABDA in the presence of ^*1*^*O*_*2*_ under continuous irradiation of white light can be described by the first-order kinetic model, where the higher first-order kinetic constant (*k*_ABDA_) indicates faster production of ^*1*^*O*_*2*_ molecules in the sample. Figure 3a shows the plasmonic enhancement effects of Au nanostars at varied concentrations on the SOG rate of AIE-PS nanodots. It was observed that for each AuNS, the singlet oxygen generation rate increases as more AuNS is added to the constant amount of AIE-PS dots until it reaches a critical concentration, beyond which the SOG rate decreases. The critical AuNS concentration with the maximum SOG is 38 pM for Au540 (*k*_ABDA_ = 0.0819 min^−1^), 38 pM for Au585 (*k*_ABDA_ = 0.0878 min^−1^), 76 pM for Au668 (*k*_ABDA_ = 0.0653 min^−1^), 152 pM for Au718 (*k*_ABDA_ = 0.0621 min^−1^), and 23 pM for Au762 (*k*_ABDA_ = 0.0805 min^−1^)-containing samples, respectively. These results show that the critical AuNS concentration is unique for each size of the studied AuNS as there is no specific trend observed, indicating the complexity of ME-SOG system, which will be studied with more details in the next section. As the amount of AIE-PS dots is fixed in all nanohybrid samples, AIE-PS dots (2 μg mL^−1^) of same concentration is used as the control sample in determining the SOG enhancement factor (*EF*_SOG_) for each AuNS@AIE-PS using the following equation:

6 $${\text{EF}}_{{{\text{SOG}}}} = \frac{{k_{{{\text{AuNS}}@{\text{AIE - PS}}}} }}{{k_{{\text{AIE - PS dot}}} }}$$

where *k*_ABDA_ is the maximum ABDA degradation rate in the presence of metal-enhanced sample ($k_{{{\text{AuNS}}@{\text{AIE - PS}}}}$) and control sample ($k_{{\text{AIE - PS dot}}}$), respectively.

Figure 3b shows the SOG enhancement factor calculated for each AuNS@AIE-PS as compared to the same amount of AIE-PS dots used in the control sample. An impressive enhancement of 15-fold for the Au585@AIE-PS sample is obtained. To the best of our knowledge, this is the highest *EF*_SOG_ value and SOG quantum yield (QY = 4.05) reported so far for the colloidal metal-enhanced singlet oxygen generation systems (see comparison of their SOG performances in Table S1) [35, 36]. To confirm the metal-enhancement effect, the SOG ability of PEI-capped AuNS was measured under the same condition as the AuNS@AIE-PS. It was found that none of the PEI-AuNSs can produce considerable amount of singlet oxygen under the same white light irradiation. Therefore, the as-observed SOG enhancement in the AuNS@AIE-PS nanohybrid is due to the plasmon enhancement effects (Fig. S3). The metal-enhanced fluorescence (MEF) effects of AuNS@AIE-PS will be discussed in the next section.

### Simulation Study and Mechanism of Metal-Enhanced Singlet Oxygen Generation

**Fig. 4:**
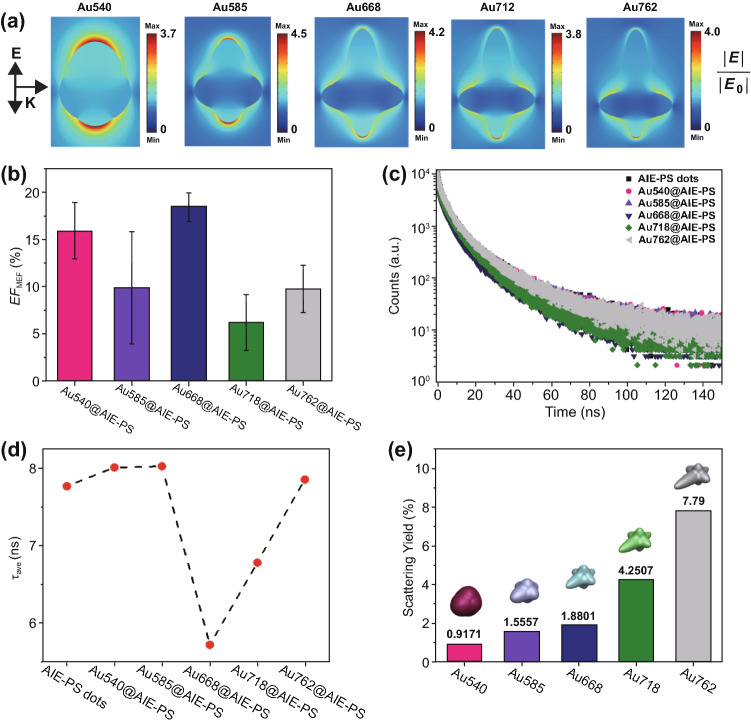
**a** Simulated enhanced electric field (|*E*|/|*E*_0_|) around different sizes of Au nanostars under 490-nm incident light, corresponding to the excitation wavelength of AIE-PS dots (i.e. 490 nm). The arrows for E and K indicate the direction of electric field polarization and incident light propagation, respectively. **b** Calculated fluorescence enhancement factor (*EF*_MEF_) for the five AuNS@AIE-PS samples at their maximum SOG, i.e. Au540@AIE-PS, Au585@AIE-PS, Au668@AIE-PS, Au718@AIE-PS, and Au762@AIE-PS (left to right). **c** Fluorescence decay. **d** Average fluorescence lifetime of different nanohybrid samples. **e** Simulated scattering yield of Au nanostars with respective LSPR peaks at 540, 585, 668, 718, and 762 nm wavelength (left to right)

To understand the origin of the observed differences in *EF*_SOG_ among the five AuNSs studied herein, we further investigated the mechanism of metal-enhanced singlet oxygen generation (ME-SOG) from an electromagnetic perspective. Simulations using the finite-element method were performed to ascertain the near- and far-field contributions to the ME-SOG. The electric field around each Au nanostar was evaluated at the excitation wavelength of the AIE-PS dots (*λ*_ex_ = 490 nm) where the maximum electric field enhancement was found in Au585 nanostar. This further confirms the experimental results as observed in the Au585@AIE-PS with the highest *EF*_SOG_ value among other nanohybrids were due to its strongly enhanced electric field, leading to a higher excitation rate in the AIE-PS nanodots and consequently more ^1^O_2_ production (Fig. 4a).

While the changes in *k*_ABDA_ values with increasing concentrations of gold nanostars (i.e. Au540, Au585, and Au762)@AIE-PS were very fast to reach the maximum SOG (> 14-fold enhancement), the samples containing Au668 and Au718 exhibited slower kinetics (Fig. 3a), respectively, resulting in lower maximum *EF*_SOG_ values. This might be due to the high energy transfer from the excited AIE-PS nanodots to these two AuNSs. To evaluate this hypothesis, we measured the fluorescence intensity as well as fluorescence lifetime for the five AuNS@AIE-PS samples at their maximum *EF*_SOG_ values (Fig. 4b, c). The metal-enhanced fluorescence enhancement factors (*EF*_MEF_) for each nanohybrid sample was calculated using the following equation:

7 $${\text{EF}}_{{{\text{MEF}}}} \left( \% \right) = \left( {1 - \frac{{{\text{Fluorescence}}\;{\text{intensity}}\;{\text{of}}\;{\text{Au}}@{\text{AIE - PS}}}}{{{\text{Fluorescence}}\;{\text{intensity}}\;{\text{of}}\;{\text{AIE - PS}}\;{\text{dots}}}}} \right) \times 100$$

As evidenced, all samples have slightly higher fluorescence intensity as compared to the AIE-PS nanodots alone. This is due to the enhanced excitation rate in AIE-PS nanodots in the vicinity of Au nanostars. However, there is no correlation found between the metal-enhancement factors for fluorescence (*EF*_MEF_) and singlet oxygen generation (*EF*_SOG_). In contrast, the average fluorescence lifetimes of Au668@AIE-PS and Au712@AIE-PS have substantially dropped, indicating the higher non-radiative energy transfer rate from the excited AIE-PS dots to the Au668 and Au718 as compared to the other nanohybrids samples (Fig. 4d). These results can be explained by the fluorescence resonance energy transfer (FRET) mechanism. As the spectral overlap for the Au668@AIE-PS and Au718@AIE-PS is higher than the other three samples (Fig. 2c), FRET occurs faster in these two nanohybrids. These results are consistent with Lovell’s findings that FRET from the excited photosensitizer molecules to other acceptor molecules results in the quenching of singlet oxygen generation [55]. However, the average fluorescence lifetime of the other nanohybrid samples, including Au540@AIE-PS, Au585@AIE-PS, and Au762@AIE-PS, is greater than that of AIE-PS dots alone, due to the strongly enhanced excitation rate as a result of the enhanced electric field around AuNSs, and thus a higher triplet state electron population in these nanohybrid samples [56, 57].

In addition, the scattering yield (i.e*.* scattering to extinction ratio) of gold nanostars with different LSPR peaks are simulated (Fig. 4e). Results show that the *EF*_SOG_ (Fig. 3b) of AuNS@AIE-PS is not proportional to the scattering yield of AuNS, which increases with a red-shift in their LSPR peaks. These results are different from Macia et al.’s finding [32], where the SOG rate of Rose Bengal could be enhanced by metal nanoparticles differently, i.e. 2.2-fold enhancement by AgNPs and lower *EF*_SOG_ values by the AuNPs and AgAu NPs of similar sizes. They have concluded that scattering yield plays an important role when metal NPs with similar electric field enhancement are being used for ME-SOG due to the alteration in the absorption cross section of photosensitizer molecule in the vicinity of plasmonic NPs. Indeed, the scattering of light by nanoparticles can provide more photons to be absorbed by the photosensitizer molecules, results in enhancement in the net absorbance of the photosensitizer molecules [16]. However, they did not consider the composition of metal core and its effect on the energy transfer rate from the excited photosensitizer molecules to the metal core in the hybrid system. Our study here has shown that the scattering yield is not the only parameter in governing the ME-SOG enhancement factor. From these results, it can be concluded that ME-SOG is a complicated phenomenon that effective factors including energy transfer, scattering yield, composition, etc., all play important roles in determining the enhancement factor.

### Cytotoxicity, Fluorescence Imaging, and Photodynamic Cancer Cell Ablation

**Fig. 5:**
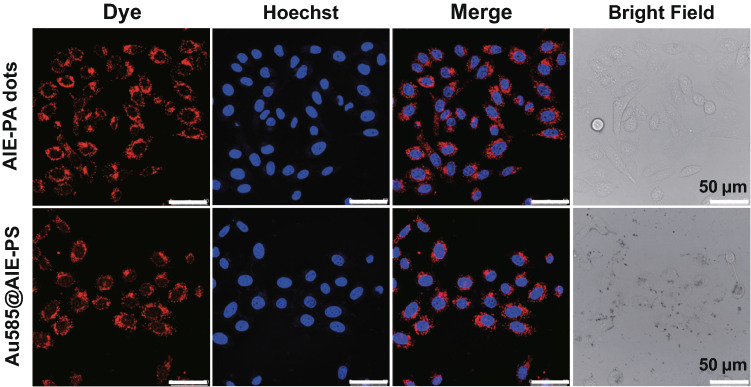
Confocal images of HeLa cells after incubation with AIE-PS dots and Au585@AIE-PS for 12 h (First column, red fluorescence). The nuclei were stained with Hoechst 33,342 (5 μg mL^−1^, 5 min) with blue emission. The difference in the contrast of bright field images (last column) indicates the internalization of Au nanostars in the cells. All the images share the same scale bar of 50 μm

The Au585@AIE-PS sample which exhibited the highest SOG enhancement among other nanohybrids was chosen for simultaneous fluorescence imaging and photodynamic cancer cell ablation. The Au585@AIE-PS sample also showed enhanced fluorescence as compared to the AIE-PS dots alone, indicating that the presence of Au585 nanostars at an appropriate concentration ratio could improve the optical properties of AIE-PS dots. The cytotoxicities of both Au585@AIE-PS and AIE-PS nanodot samples were evaluated using MTT viability assay on the NIH-3T3 mammalian cells and HeLa cancer cells (Fig. S4a, b). The results show that Au585@AIE-PS and control samples (AIE-PS nanodots) possess very low dark cytotoxicity towards both cell lines even at high concentration of 50 µg/mL (based on AIE-PS mass concentration). The presence of Au nanostars has a negligible effect on the cytotoxicity of nanohybrid sample. Figure 5 shows the confocal laser scanning microscopy (CLSM) images of HeLa cells after incubation with AIE-PS dots and Au585@AIE-PS samples (10 µg mL^−1^) for 12 h. The bright red fluorescence images originated from the AIE-PS nanodots were observed in the cytoplasm in HeLa cells where the cell nucleus was stained with the Hoechst 33,342 dye with blue emission. These results clearly demonstrated the ability of using the as-developed Au585@AIE-PS for bright fluorescence imaging.

**Fig. 6:**
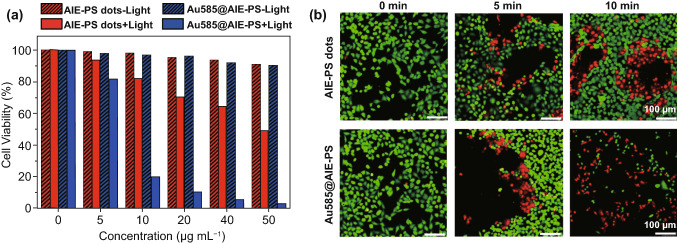
**a** Cell viabilities of AIE-PS dots and Au585@AIE-PS nanohybrids treated HeLa cells with ( +) or without (−) white light irradiation (100 mW cm^−2^, 10 min) provided by the LB-150 Cold Light Illumination. Control studies showing cell viability in the presence of respective samples under dark conditions (pattern-filled). **b** Live/dead staining of AIE-PS dots and Au585@AIE-PS nanohybrids treated HeLa cells with varied light irradiation times (0, 5, and 10 min) at a power of 100 mW cm^−2^. The live cells were stained by fluorescein diacetate (green, 50 μg mL^−1^ for 10 min), whereas dead cells were stained by PI (red, 100 μg mL^−1^ for 10 min). The excitation wavelength for the green fluorescein diacetate, and the red fluorescence PI is 488 and 543 nm, respectively

The in vitro photodynamic therapy (PDT) experiments were conducted using the red fluorescent Au585@AIE-PS nanohybrids and AIE-PS nanodot (as control) upon 12 h of incubation with the HeLa cancer cells, followed by 10 min of light irradiation (100 mW cm^−2^). The efficiency of PDT was evaluated by the standard MTT assay. As shown in Fig. 6a, although both the Au585@AIE-PS and AIE-PS dots showed very low dark cytotoxicity (< 3% cell death) at the tested concentration (i.e., 10 µg mL^−1^ based on photosensitizer mass), a huge difference in cancer cell viability was observed under white light treatment. That is, more than 80% of HeLa cells incubated with the Au585@AIE-PS were killed in 10 min, while only 18% cell death was found in the cellular sample with AIE-PS dots alone under the same PDT conditions. This significant difference in the cell viability shows the enhanced singlet oxygen generation of AIE-PS due to the plasmonic effect of Au585 nanostars. In addition, MTT assay was performed on the Au585 nanostars (with and without light) under the exact same condition of incubation and light irradiation as that of the Au585@AIE-PS nanohybrids. The results in Fig. S4c show that gold nanostars at various concentrations have no phototoxicity on HeLa cells, further confirming that the enhanced phototoxicity as-observed in this study is indeed due to the plasmonic effect of Au585 nanostars. Additionally, we have compared the phototoxicity of the as-developed Au585@AIE-PS nanohybrid with Verteporfin as a model commercial photosensitizer. Results show that the nanohybrid possesses comparable PDT performance with clinically used photosensitizer under similar light treatment conditions (Fig. S4d). Furthermore, it was found that both the AIE-PS nanodots and Au585@AIE-PS nanohybrid possess much higher photostability as compared to the three different commercial photosensitizers (i.e. Verteporfin, Rose Bengal, and Methylene Blue). As shown in Fig. S4e, the presence of Au585 can further improve the photostability of AIE-PS in the nanohybrids, which is one of the advantages of the metal-enhanced systems as demonstrated herein [16]. Additionally, the as-developed Au585@AIE-PS nanohybrid showed excellent anti-photobleaching property (Fig. S4f), which is extremely useful for image-guided therapy applications.

To directly visualize the PDT effects, we also performed the live/dead staining of the HeLa cells with fluorescein diacetate (green for live cells) and propidium iodide (red for dead cells) in the presence of Au585@AIE-PS and control sample under different light irradiation time (Fig. 6b). Under dark conditions, bright green fluorescence with no red emission was observed in cellular images, indicating the excellent biocompatibility of both Au585@AIE-PS and AIE-PS nanodots (control). In contrast, along with the increase in light irradiation time, the population of green fluorescent (live) cells decreased, while the population of red fluorescent (dead) cells increased. As can be seen, the ratio between the red to green fluorescent cells is much higher in the samples incubated with the Au585@AIE-PS, showing that HeLa cancer cells can be effectively killed by the Au585@AIE-PS upon light irradiation in less than 10 min.

## Conclusions

In this work, we have successfully developed the red-emissive Au nanostar@AIE photosensitizer nanodots with greatly enhanced singlet oxygen generation and fluorescence brightness for simultaneous photodynamic therapy and imaging of HeLa cancer cells. Five different Au nanostar@AIE-PS nanodots were prepared in this study by varying the core size of Au nanostar, allowing to tune the degree of spectral overlap between the extinction of AuNS and fluorescence of AIE-PS. Both the AuNS concentration and their spectral overlap with the fluorescence of AIE-PS determined the SOG enhancement factor (*EF*_SOG_) of AuNS@AIE-PS. In particular, a 15-fold enhancement was found in the AuNS@AIE-PS sample containing 38 pM of Au585 nanostars (i.e. with LSPR peak at 585 nm) and 2 µg mL^−1^ AIE-PS dots. Simulation studies have further confirmed the critical role of the electric field in Au585 nanostars to promote the intrinsic SOG of AIE-PS. In addition, it was noticed that larger spectral overlap resulted in lower *EF*_SOG_ value. Interestingly, fluorescence enhancement instead of quenching was observed in all the AuNS@AIE-PS samples, which is an extra benefit for fluorescence imaging. The Au585@AIE-PS nanohybrids with low dark cytotoxicity were then applied for simultaneous fluorescence imaging and cancer cell ablation. It was found that > 80% of HeLa cells were killed in 10 min, while only 18% cell death was found in the control sample (e.g. AIE-PS) under the same PDT conditions. This study clearly demonstrates an efficient approach to enhance the singlet oxygen generation of AIE photosensitizers without fluorescence quenching using biocompatible Au nanostars that could be served as promising multifunctional photosensitizers toward effective image-guided photodynamic therapy in future nanomedicine.

## Supplementary Information

Below is the link to the electronic supplementary material.

Supplementary Information (DOCX 2220 kb)
