# Supplementary material for: Gold Nanostars-AIE Theranostic Nanodots with Enhanced Fluorescence and Photosensitization Towards Effective Image-Guided Photodynamic Therapy
Source: Nanomicro Lett. 2021 Jan 16;13:58. doi: 10.1007/s40820-020-00583-2 (PMC8187487; doi:10.1007/s40820-020-00583-2)
Supplement: Supplementary file 1 — Supplementary Information (DOCX 2220 kb) [file 40820_2020_583_MOESM1_ESM.docx]

**Supporting Information**

**Gold nanostars-AIE theranostic nanodots with enhanced fluorescence and photosensitization towards effective image-guided photodynamic therapy**

Mohammad Tavakkoli Yaraki^1,2^, Min Wu^2,✝^, Eshu Middha^2,✝^, Wenbo Wu^2^, Soroosh Daqiqeh Rezaei^1,3^, Bin Liu^2,*^, Yen Nee Tan^1,4,5,*^

^1^ Institute of Materials Research and Engineering, Agency for Science, Technology and Research (A*STAR), 138634, Singapore

^2^ Department of Chemical and Biomolecular Engineering, National University of Singapore, 4 Engineering Drive 4, Singapore 117585, Singapore

^3^ Department of Mechanical Engineering, Faculty of Engineering, National University of Singapore, 9 Engineering Drive 1, 117575, Singapore

^4^ Faculty of Science, Agriculture & Engineering, Newcastle University, Newcastle Upon Tyne NE1 7RU, United Kingdom

^5^ Newcastle Research and Innovation Institute (NewRIIS), 80 Jurong East Street 21, #05-04, Singapore 609607

*Corresponding authors. A/Prof. Yen Nee Tan, E-mail: [yennee.tan@newcastle.ac.uk](mailto:yennee.tan@newcastle.ac.uk); Prof. Bin Liu, E-mail: [cheliub@nus.edu.sg](mailto:cheliub@nus.edu.sg)

^✝^M.W. and E.M. contributed equally to this work.

**SI-1 Microfluidic synthesis of ultra-small AIE-dots**


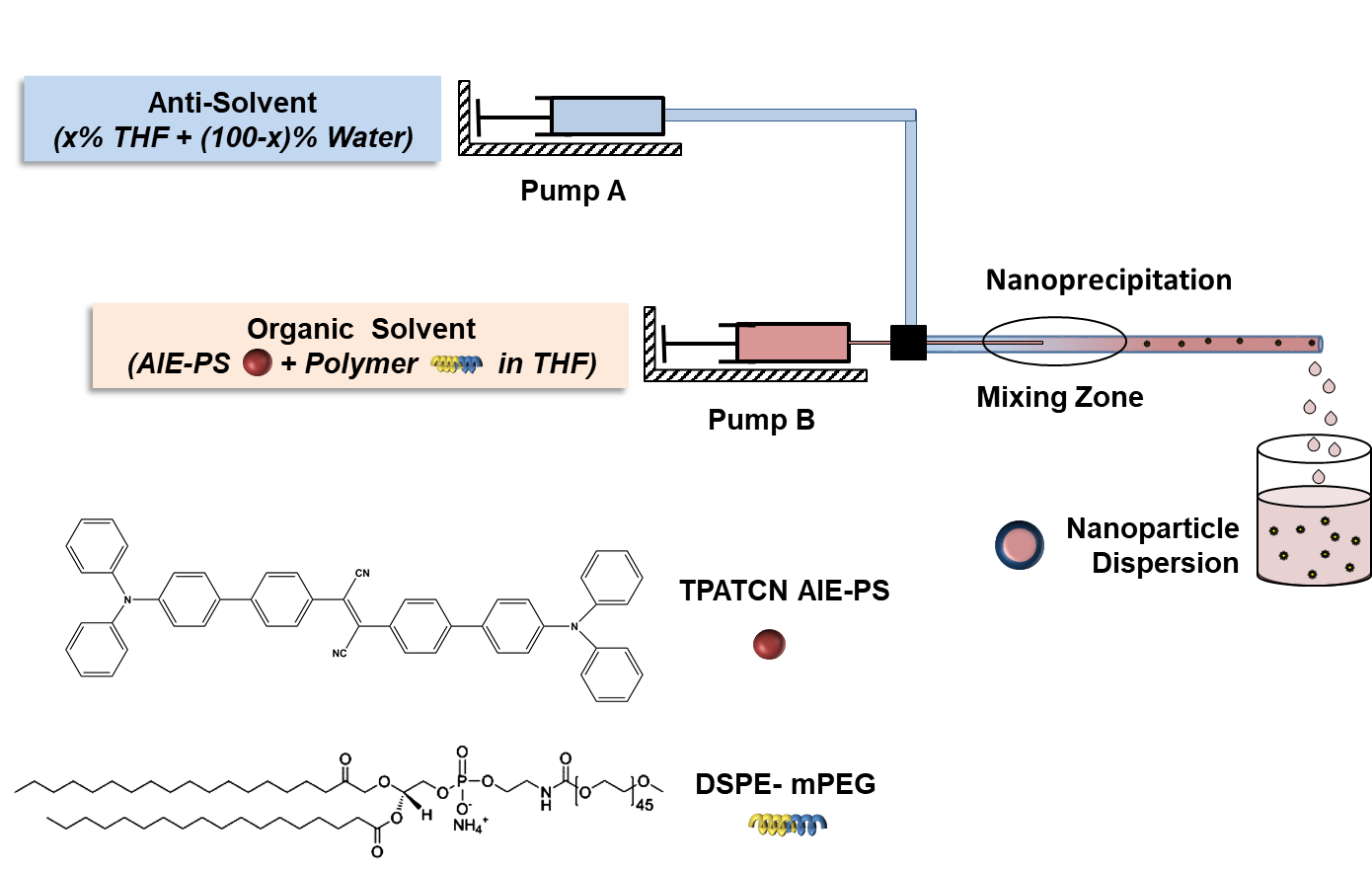


**Scheme S1**. Schematic of the microfluidic glass capillary mixer for the synthesis of monodisperse TPATCN-loaded DSPE–mPEG2000 NPs.

Pump A controls the flow of anti-solvent (outer flow), which contains water. On the other hand, syringe pump B controls the flow rate of organic solvent acetone (inner flow) with polymer and ROS dye dissolved in it. To maintain the high level of supersaturation during mixing, the volumetric flow rate ratio of outer to inner fluid is maintained as 10. The solvent stream comprises of ROS dye and DSPE-mPEG with a polymer to dye ratio (PDR) of 2. The inner fluid (solvent) is rapidly mixed with outer fluid (anti-solvent) in the mixing zone, resulting in NPs through the self-assembly process. Then, the mixture is kept for solvent evaporation. The final concentration of AIE-dots was 0.3 mg/mL.

***SI.1.1. Fabrication of Microfluidic mixer***

The coaxial flow microfluidic glass capillary mixer was assembled by using two round glass capillaries of diameter 1 mm (for outer) and 0.7 mm (for inner), Teflon tubings and fittings from Chemikalie Pte Ltd. The inner round capillary was pulled using a micropipette puller (Sutter Instruments, P-97) to produce a tapered end. The tapered end was then enlarged to 100 µm using abrasive paper. Teflon tubing was used to connect the glass capillary device to respective syringes controlled by syringe pumps. The inner glass capillary was inserted into the outer glass capillary with a side hole in Teflon tubing. The outer glass capillary is connected with a plastic Monoject syringe through Teflon tubing and fittings for the supply of water. The pump used for the water supply was a Chemyx Nexus-3000 pump, and the organic phase was injected by using a Chemyx Fusion-200 pump.

***SI.1.2. Calculation of Reynolds number and flow velocity***

The total flow rate in the system can vary from 1.1 mL min^-1^ to 16.5 mL min^-1^. The relation between flow velocity (u) and volumetric flow rate (Q) is defined by Equation ‎S1.

$u=\frac{4Q}{\pi D_{i}}$  Equation S1

where D_i_ is the inner diameter of the pipe (1 mm)

Re in the system was varied on the basis of the total flow rate using the following Equation ‎S2.

$Re=\frac{\rho uL}{\mu}$ Equation S2

where μ represents the viscosity of the fluid, ρ represents the density of the fluid, and L is a characteristic linear dimension (m). For a circular pipe, the characteristic linear dimension is the same as the hydrodynamic diameter (inner diameter of the pipe). Equation S3 represents the relation between Re and flow rate.

$Re=\frac{4\rho Q}{\pi\mu}$ Equation S3

**Figure S1** shows the variation of Re with respect to flow rate:


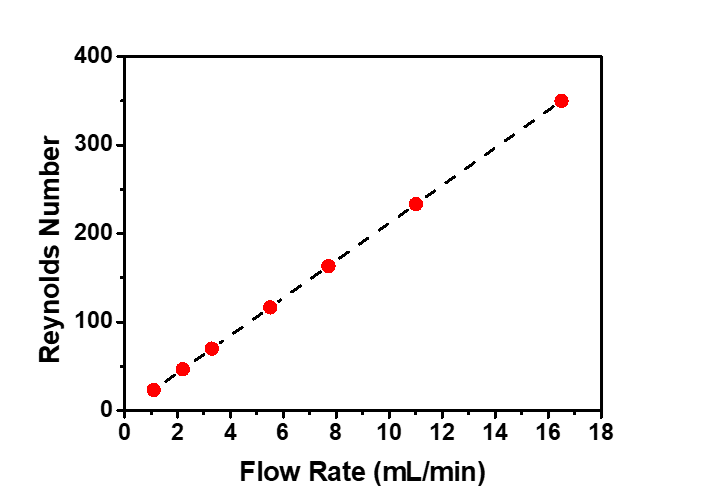


**Figure S1**. Variation of Re in the pipe due to the change in flow rate (mL min^-1^)


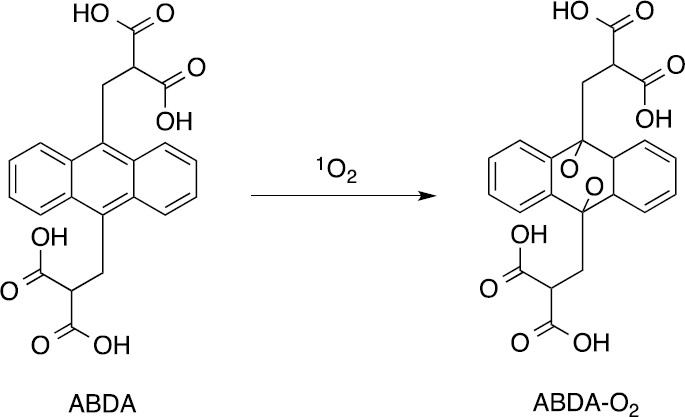


**Scheme S2**. Reaction of ABDA with ^1^O_2_ to generate an endoperoxide, referred to as (ABDA-O_2_)

**
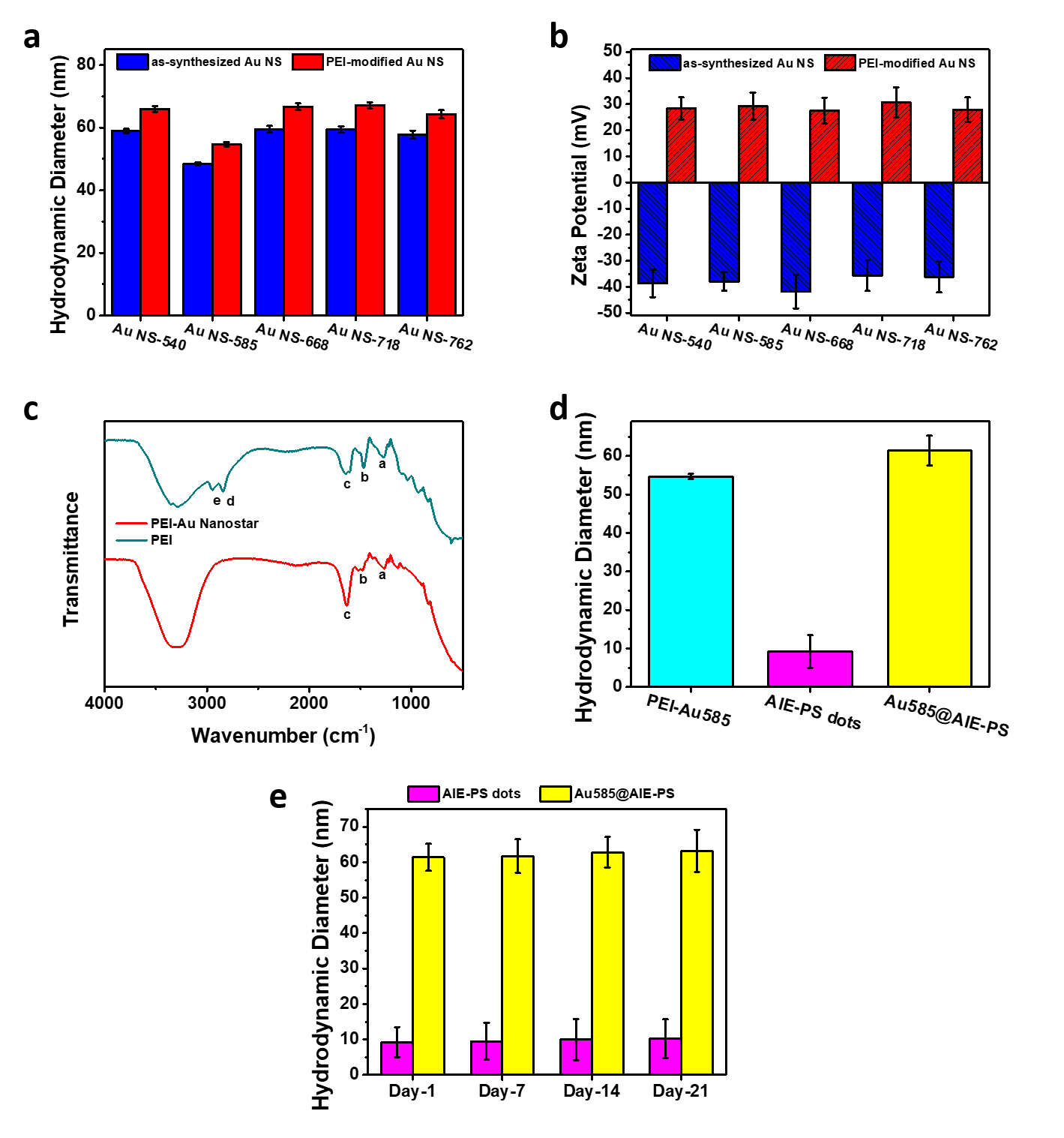
**

**Figure S2.** a) Hydrodynamic diameter and b) Zeta potential value of the as-synthesized AuNSs and PEI-modified AuNSs. A slight increase in the size and also change in the zeta potential values from negative to positive indicate the successful surface modification of AuNSs by PEI. c) FTIR spectra of PEI-modified AuNSs and PEI solution. The characteristic vibrations of PEI (a: C-N stretching vibrations of the tertiary amine, b: coupling of N-H angular deformation and C-N stretching vibrations, c: scissoring vibration of –NH2 in PEI) are appeared in the PEI-modified AuNSs, indicating successful surface coverage of AuNSs by PEI. Other vibrations (d: symmetric stretching of C-H, e: asymmetric stretching of C-H) are merged with a strong vibration of OH groups due to the presence of water in PEI-AuNSs sample. d) Hydrodynamic diameter of PEI-Au585, AIE-PS dots, and Au585@AIE-PS nanohybrid. e) The changes in the hydrodynamic diameters of AIE-PS dots and Au585@AIE-PS nanohybrid during storage at temperature of 4 ^o^C.

**Table S1.**Comparison of singlet oxygen generation (SOG) performances of different reported metal nanoparticles-enhanced SOG systems

| **Nanohybrid** | ***Enhancement Factor (EF*_SOG_)** | **SOG Quantum Yield (QY)** | **Reference** |
| --- | --- | --- | --- |
| AgNPs@Silica-Rose Bengal | 3-fold | 2.25 | [1] |
| AgNanocube@Silica-Rose Bengal | 4-fold | 3.00 | [2] |
| SiO2@Ag@SiO2‑Rose Bengal | 1.32-fold | 0.99 | [3] |
| Au585@AIE-PS | 15-fold | 4.05 | This work |

**References**

1. O. Planas, N. Macia, M. Agut, S. Nonell, B. Heyne. Distance-dependent plasmon-enhanced singlet oxygen production and emission for bacterial inactivation. Journal of the American Chemical Society. **138**(8), 2762-2768 (2016).

2. N. Macia, R. Bresoli-Obach, S. Nonell, B. Heyne. Hybrid silver nanocubes for improved plasmon-enhanced singlet oxygen production and inactivation of bacteria. Journal of the American Chemical Society. **141**(1), 684-692 (2019).

3. V. Kabanov, B. Heyne. Impact of incoherent coupling within localized surface plasmon resonance on singlet oxygen production in rose bengal-modified silica-coated silver nanoshells (sio2@ag@sio2-rb). ACS Applied Nano Materials. **3**(8), 8126-8137 (2020).


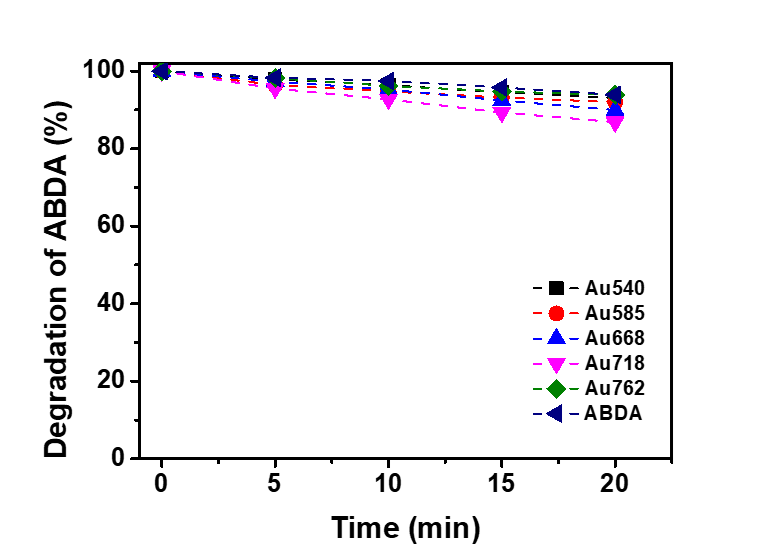


**Figure S3**. Degradation of 50 μM ABDA in the presence of different Au nanostars (304 pM) under 30 mW/cm^2^ white light


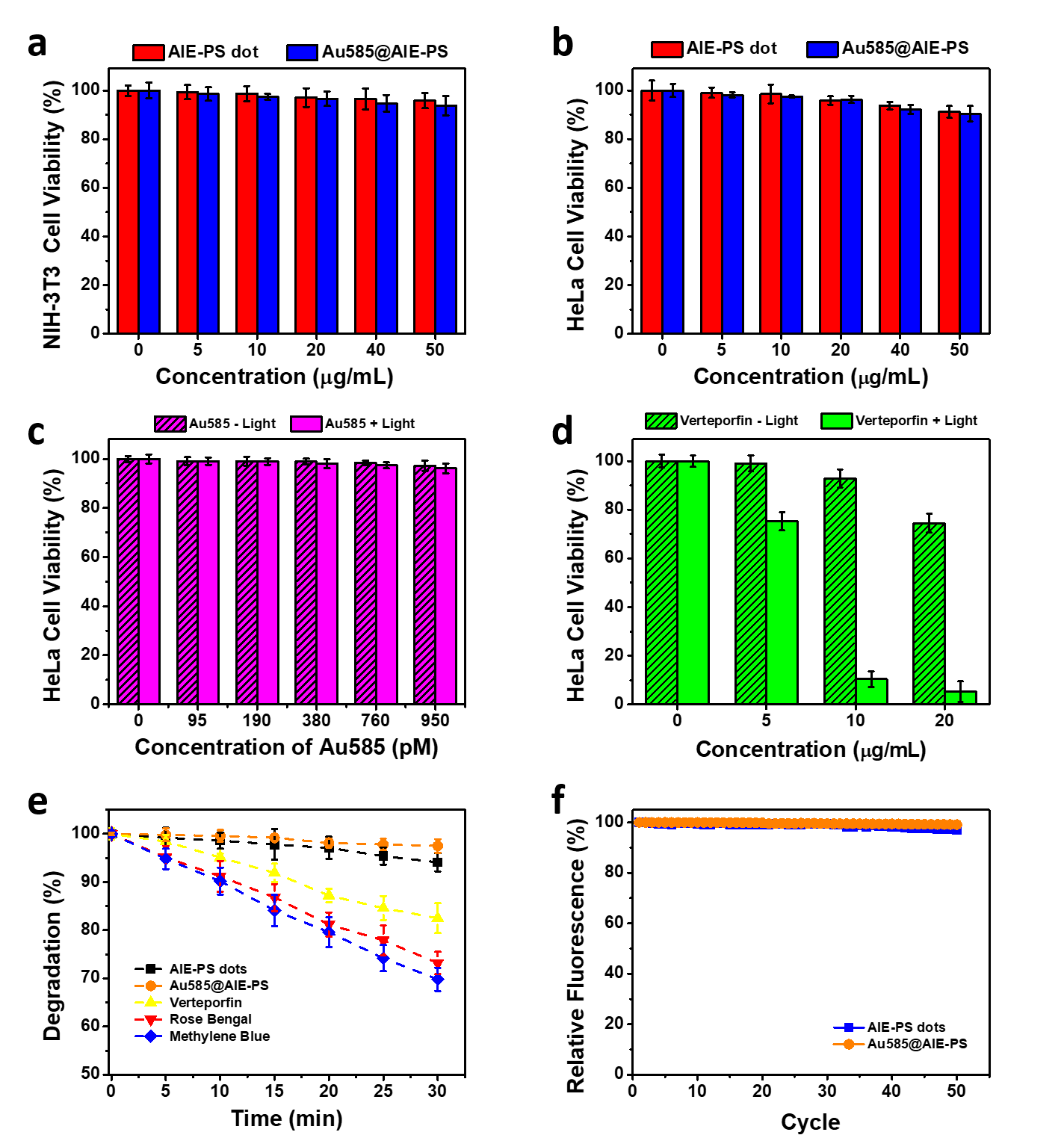


**Figure S4**. Metabolic viability of NIH-3T3 cells (a) and HeLa cells (b) as function of AIE-photosensitizer concentration (0-50 µg/mL), after 12 hr incubation with AIE-PS dot (red) and Au585@AIE-PS (blue), respectively. Metabolic viability of HeLa cells as function of Au585 nanostars concentration (c) and Verteporfin concentration (d) after 12 hr incubation. All conditions were similar for (a-d) and the concentration of Au585 nanostars was chosen according to their concentrations in the Au585@AIE-PS nanohybrid. For the Au585@AIE-PS sample, the concentration ratio of Au585 to AIE-PS dots was fixed at 38 pM : 2 µgmL^-1^. (e) The photostability of AIE-PS dots, Au585@AIE-PS, Verteporfin, Rose Bengal and Methylene Blue after illumination (white light, 100 mW/cm2). f) Excellent photobleaching property of AIE-PS dots and Au585@AIE-PS nanohybrid monitored for up to 50 cycles under excitation wavelength at 490 nm

**
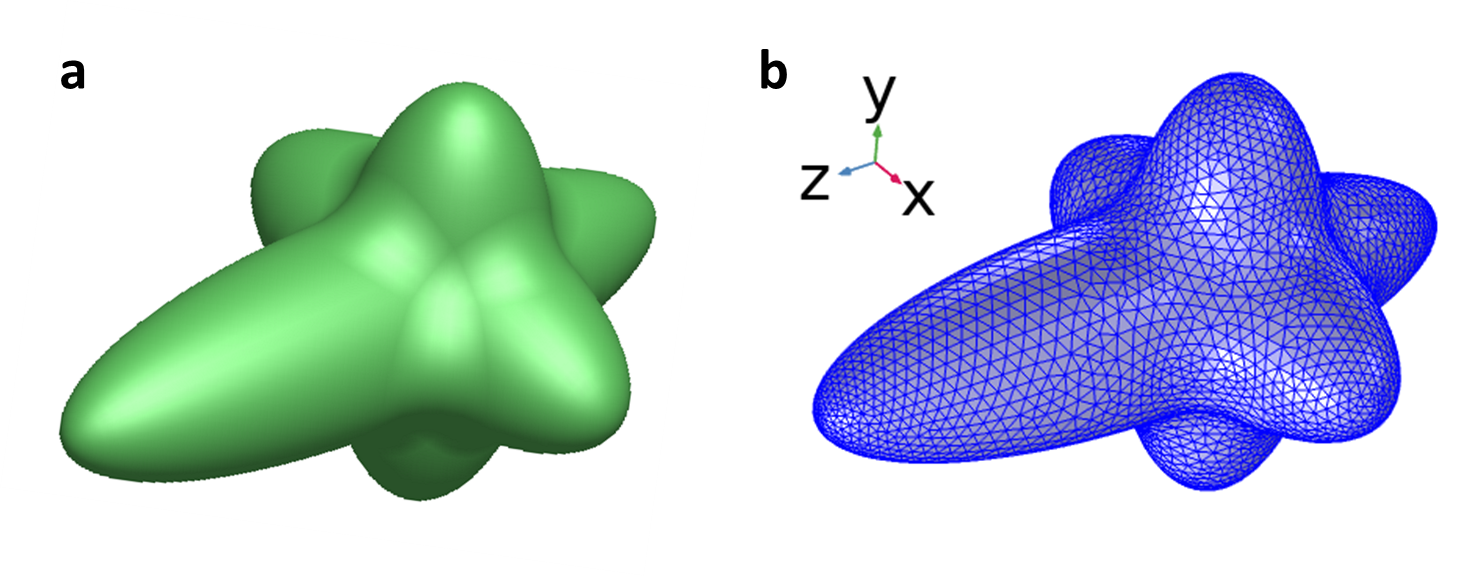
**

**Figure S5**. Example nanostar modeled in SolidWorks (a) and then meshed in Comsol Multiphysics

**Table S2.** Size analysis of different Au nanostars and the corresponding experimental and simulated SPR peaks

| **Sample** | **Short axis (nm)** | **Middle axis**  **(nm)** | **Long axis**  **(nm)** | **Eccentricity**  **(nm)** | **Experimental SPR Peak**  **(nm)** | **Simulated**  **SPR Peak**  **(nm)** |
| --- | --- | --- | --- | --- | --- | --- |
| Au540 | 15 | 18 | 19 | 21 | 540 | 540 |
| Au585 | 13 | 21 | 22 | 27 | 585 | 590 |
| Au668 | 8 | 18 | 19 | 25 | 668 | 670 |
| Au718 | 12 | 27 | 29 | 42 | 718 | 720 |
| Au762 | 18 | 30 | 38 | 56 | 762 | 765 |


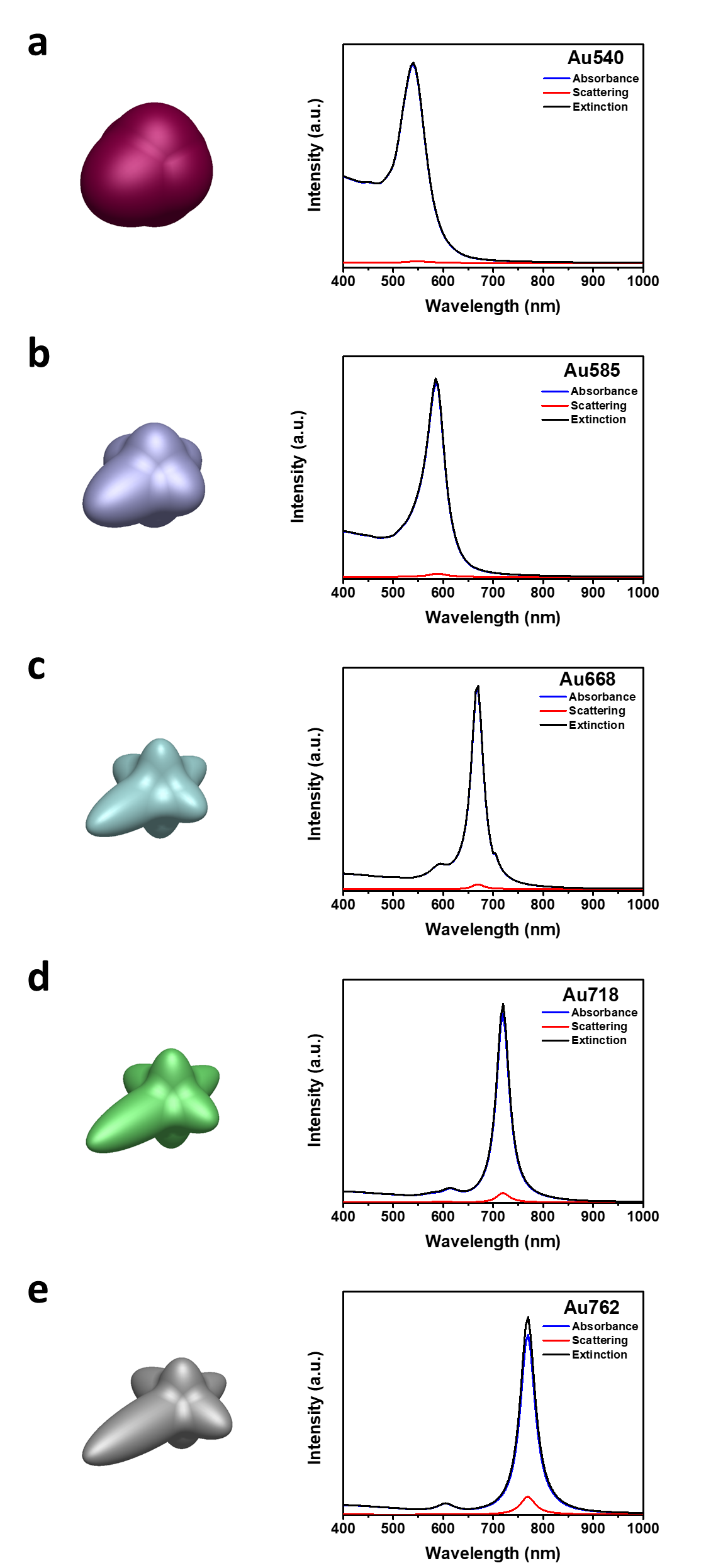


**Figure S6.** Simulated absorbance, scattering, and extinction spectra for different Au nanostars: a) Au540, b) Au585, c) Au668, d) Au718, and e) Au762.
